# Real-Time Abnormal Event Detection for Enhanced Security in Autonomous Shuttles Mobility Infrastructures

**DOI:** 10.3390/s20174943

**Published:** 2020-09-01

**Authors:** Dimitris Tsiktsiris, Nikolaos Dimitriou, Antonios Lalas, Minas Dasygenis, Konstantinos Votis, Dimitrios Tzovaras

**Affiliations:** 1 Information Technologies Institute, Centre for Research and Technology Hellas, 6th km Charilaou-Thermi, 57001 Thermi, Greece; tsiktsiris@iti.gr (D.T.); nikdim@iti.gr (N.D.); kvotis@iti.gr (K.V.); dimitrios.tzovaras@iti.gr (D.T.); 2 Department of Electrical and Computer Engineering, University of Western Macedonia, 50100 Kozani, Greece; mdasyg@ieee.org

**Keywords:** computer vision, deep learning, autonomous shuttles, petty crimes detection, smart mobility

## Abstract

Autonomous vehicles (AVs) are already operating on the streets of many countries around the globe. Contemporary concerns about AVs do not relate to the implementation of fundamental technologies, as they are already in use, but are rather increasingly centered on the way that such technologies will affect emerging transportation systems, our social environment, and the people living inside it. Many concerns also focus on whether such systems should be fully automated or still be partially controlled by humans. This work aims to address the new reality that is formed in autonomous shuttles mobility infrastructures as a result of the absence of the bus driver and the increased threat from terrorism in European cities. Typically, drivers are trained to handle incidents of passengers’ abnormal behavior, incidents of petty crimes, and other abnormal events, according to standard procedures adopted by the transport operator. Surveillance using camera sensors as well as smart software in the bus will maximize the feeling and the actual level of security. In this paper, an online, end-to-end solution is introduced based on deep learning techniques for the timely, accurate, robust, and automatic detection of various petty crime types. The proposed system can identify abnormal passenger behavior such as vandalism and accidents but can also enhance passenger security via petty crimes detection such as aggression, bag-snatching, and vandalism. The solution achieves excellent results across different use cases and environmental conditions.

## 1. Introduction

The deployment of autonomous vehicles (AVs) can provide benefits such as cost reductions along with improving accessibility to transportation services via decreased travel costs. However, there are some end users’ concerns, regarding the Safety and Robustness of the AVs. The prospective passengers fear several possible instances that could arise in case there is no driver in the bus. Among them, there is the feeling of discomfort being all alone in the bus at night, especially in certain neighborhoods, the fact that no authority figure is present to keep passengers calm or to perform first aid if required and handle abnormal events such as vandalism, etc. To address the aforementioned concerns on social and personal safety and security in the vehicle, certain measures need to be implemented. A solution for enhancing the safety and security inside the autonomous buses will support protecting passengers and safekeeping the vehicles.

Automatic awareness of human actions and their interaction with the environment has become a prominent study area in recent years. To perform such a demanding mission, many scientific areas rely on modeling human activity in its various dimensions (emotions, relational attitudes, actions, etc.). In this context, the identification of a person’s activity tends to be necessary to the comprehension of specific acts. Thus, human action recognition has gained a lot of interest, especially in real-world environments. So far, several methods are based on multiple dimension pose datasets and color frame sequences in order to recognize actions. Methods based on colored videos [1,2,3,4] typically model spatiotemporal features from frame sequences in conjunction with temporal optical flow. In spite of their positive results, color frame sequences still have limitations stemming from background perplexity, changes in lighting and appearance, and other issues. To mitigate those restrictions, multidimensional skeleton data are used for displaying the human body with a series of 3D key-points. Due to the lack of RGB channels, those restrictions do not apply in sequences with skeleton data. Such robustness allows to model more distinctive human actions characteristics. The fact that key joints offer highly useful information about human motion is a conceptual basis provided by Johansson et al. [5]. Apart from Microsoft Kinect [6], there are also many advanced algorithms for human pose estimation [7] that are capable of acquiring skeleton data. Methods used to recognize skeleton-based actions examine various models to learn spatiotemporal features. A research by Song et al. [8] developed a spatiotemporal Long Short-Term Memory (LSTM)-based model for distinctive spatiotemporal features. Another method appropriate for feature learning is the convolutional neural network (CNN), as presented in [9,10,11]. In addition, Yan et al. [12] used graph convolutional networks (GCN), suggesting exploiting both LSTM and graph neural networks to represent temporal and spatial data, respectively. In brief, all these aforementioned methods aim to design an efficient model for identifying spatiotemporal skeleton features. However, effective distinctive feature extraction is considered as a significant challenge.

In this paper, various techniques were implemented and compared for the timely, accurate, robust, and automatic detection of various petty crime types as well as for the assistance of authorized end-users towards the re-identification of any offenders. The proposed solution can identify abnormal passenger behavior such as vandalism and accidents (e.g., passenger falling) but can also enhance passenger security via petty crimes detection (aggression, bag-snatching). In this research, the goal is to define an appropriate solution, able to address such complications using modern technologies and deep learning techniques. The solution:

- supports end-to-end detection of abnormal events,
- achieves real-time inference on modern hardware,
- offers flexibility with supervised, unsupervised and semi-supervised learning, to compensate the scarcity of data in the security domain,
- supports multiple camera types, positions and angles,
- is able to operate in embedded setup with limited power requirements.

In Section 2, we attempt a brief overview of the most relevant methods to our work. Section 3 provides details on the datasets used for model training and evaluation. In Section 4, we explain the methodology along with the design principles, techniques involved and critical components of the system. Finally, Section 5 illustrates the experimental environment and the results of the proposed solution.

## 2. Related Work

Mahadevan et al. [13] proposed an anomaly detection framework appropriate for crowded scenes. According to their research, they were able to detect anomalies in clustered scenes using joint modeling of scene dynamics and appearance, as well as abilities of discovering spatial and temporal abnormalities. The normal crowd behavioral model is based on combining dynamic textures, whereas outliers in it are identified as anomalies. Spatial anomalies are handled using discriminant saliency, while temporal anomalies are associated to low probabilistic events. For the experimental evaluation they used crowded scenes as a dataset, utilizing 100 video sequences and five well-defined abnormality categories. Avilés et al. [14] presented a coarse-fine convolutional method for human activity recognition using smartphone sensors. Ordóñez et al. [15] have also proposed techniques for wearable human activity recognition using multimodal deep convolutional and LSTM recurrent neural networks. Another interesting study from Sabokrou et al. [16] presented an effective framework appropriate for anomaly detection and localization in video streams. Using both temporal data and fully convolutional neural networks (FCNs), they proposed a pre-trained supervised FCN, that later on transfers into an unsupervised FCN, capable of detecting global anomalies. By investigating the cascaded detection resulting in computation complexities reduction, the model offers high accuracy and speed. This FCN-based architecture can be applied in videos to represent features and detect cascaded outliers. Yuan et al. [17] proposed a structural context descriptor (SCD) that describes the crowd individual, initially introducing the potential energy function of particles’ interforce in solid-state physics for intuitive contextual cueing of vision. So as to compute the crowd SCD variation successfully, they designed a robust multi-object tracker, employing the incremental analytical ability of the 3D discrete cosine transform (DCT) to associate the targets in different frames. With the proposed method, the abnormality is localized with the online spatiotemporal analysis of the crowd SCD variation. Fernando et al. presented an LSTM framework that fits for both predicting human trajectories and detecting abnormal events using deterministic models called soft attention models, which are trained using back-propagation [18]. Ravanbakhsh et al. [19] combine semantic information (inherited from existing CNN models) with low-level optical-flow, in order to measure local abnormality. Wei et al. [20] achieved a more robust human action recognition. Firstly, they combined video images with simultaneously captured inertial signals, using a video camera and a wearable inertial sensor within a fusion framework, and then turned them into 3D video and 2D inertial images. Finally, once these images are fed as inputs into a 3D convolutional neural network and a 2D convolutional neural network, respectively, the model is able to perform action recognition. Other approaches focused on the transport domain, such as [21,22,23,24], rely on facial expressions and behavior, gaze and eye tracking for detecting emotions, stress, anxiety, and panic. Although effective, those methods add another layer of complexity to our solution, as they require high resolution facial images which subsequently introduce privacy issues.

Most of the aforementioned techniques are heavily tuned in the surveillance of large areas, while others are using multiple input from other sensors (e.g., wearables, accelerometers, depth sensors etc.), which are not available in our application as we rely only on 2D data from the camera sensors. Trajectory-based methods are providing limited results, when applied to camera with restricted field of view, since heavy preprocessing (e.g., tracking, occlusion detection, joint reconstruction) is needed to eliminate false information which may confuse the model. In addition, as mentioned in Section 4, one of the design constraints is the support of multiple camera types including wide-angle lens with higher field of view. Although the classification via pose estimation can provide excellent results in regular camera lens, fundamental issues are identified in top-down views. In some cases, the whole body of a passenger is occluded by the head, rendering the pose estimation impossible. For such applications, techniques based on regularity learning were employed. Encodings based on spatial information (convolutional autoencoders) are not aware of time and, therefore, cannot identify actions and events. Spatiotemporal models are the most promising for our application, but they are not robust enough to be used as a standalone solution. Therefore, we propose an end-to-end solution based on camera sensors and deep learning techniques, able to operate in a embedded environment inside the autonomous vehicle and perform online inference using three different approaches: (a) a Stacked Bidirectional LSTM Classifier, (b) a Spatiotemporal Autoencoder, and (c) a Hybrid LSTM Classifier. A summary of each method’s strengths and drawbacks is presented in Table 1.

## 3. Dataset

There is a restrained tendency on sharing data related to security applications. Surveillance data are considered sensitive and related to confidential and legal issues. Moreover, the few available data are often tailored to a specific application and require adaptations in order to be useful. The methods and experiments we developed require a large amount of data due to the nature of the machine learning algorithms. To overcome this obstacle, we performed several data capture sessions on both simulated and real-world scenarios. Therefore, our solution is based mostly in our own data in order to be accurate and satisfy the design constraints. In addition, based on some techniques for data augmentation and methods associated to weak supervision, this issue was sufficiently mitigated.

The petty crimes that are targeted for identification by the sensors include petty theft like bag-snatching and pickpocketing, vandalism and aggression. While a commuter is in the autonomous shuttle a petty crime takes place in the form of assault. The commuter is attacked by another person (Use Case 1: Fighting/Aggression) who is attempting to snatch his bag (Use Case 2: Bag-Snatching). The aggressive incident is captured by the sensors in the shuttle and fed to the video analytics component for further analysis. At a later stage, the system is capable of sending a security alert to the operator or security supervisor. The course of action of the operator is a human decision, that means, whether he/she will decide to stop the autonomous shuttle or will notify the passengers via the radio system. A third use case focuses on the detection of vandalism (Use Case 3: Vandalism). A young person boards the autonomous shuttle during an itinerary performed by the vehicle during the night hours. The person attempts to perform a vandalism action on the shuttle, through painting graffiti or trying to smash the windows. The night mode of the cameras installed in the vehicle acquire the data that will be fed to the video analytics algorithms for further analysis. The person is warned by the radio system of the autonomous shuttle or security personnel intervenes by stopping the bus.

**Figure 1:**
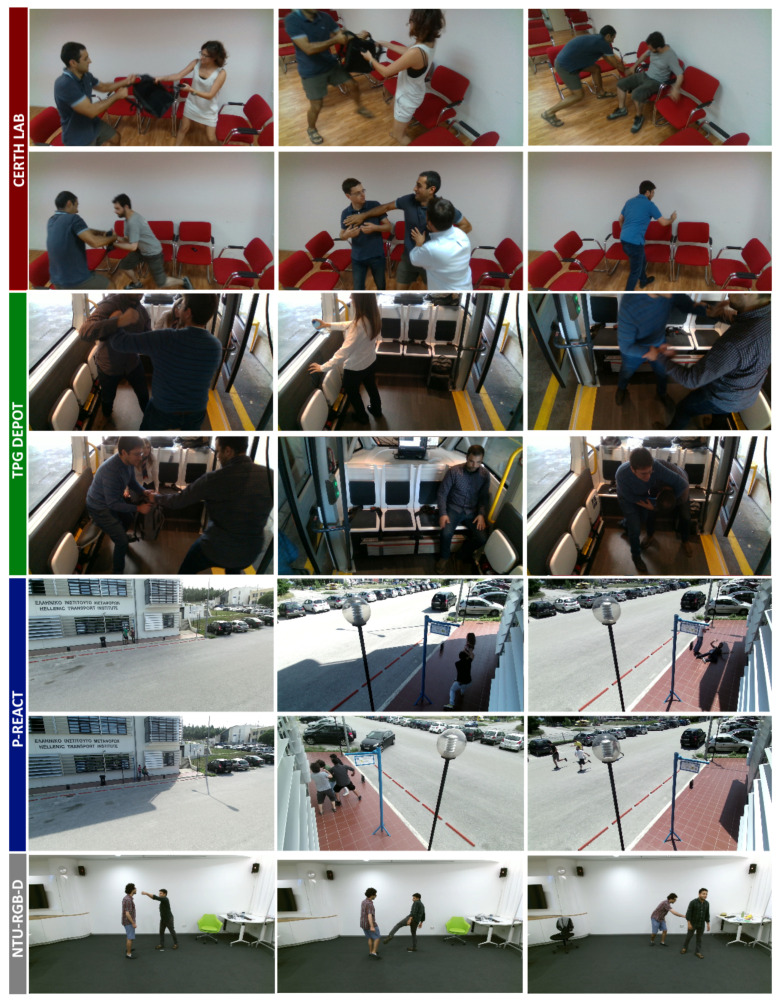
Dataset samples with abnormal events which showcase the use cases. Fighting, aggression, bag-snatching, and vandalism scenarios are illustrated. The red section contains simulated data in lab, green section depicts captured data from TPG shuttles, blue section shows scenarios from P-REACT dataset, and the gray section indicates additional data imported from the NTU-RGB dataset.

**Figure 2:**
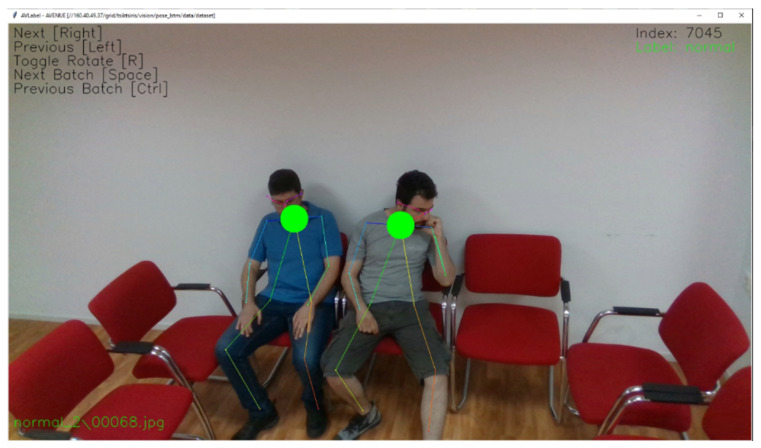
VLabel: Pose labeling using mouse clicks on the skeleton’s circle and keyboard navigation.

In this paper, five datasets were used: (a) data simulated in lab, (b) data captured from Geneva Public Transport (TPG) shuttles, (c) from petty criminality diminution through search and analysis in multi-source video capturing and archiving platform (P-REACT) [25] project, (d) the NTU-RGB-D [26] dataset by ROSE lab, and (e) the UCSD Anomaly Detection dataset [13]. An overview of those datasets is illustrated in Figure 1. As an initial approach, we conducted several recordings on our labs at the Centre of Research and Technology Hellas Information Technologies Institute (CERTH/ITI) facilities, simulating the shuttle environment as close as possible. In this session, 13 different scenarios were simulated using two different camera perspectives for each one. The dataset contains 6650 frames that, in conjunction with some augmentation techniques described later, were sufficient to train the LSTM Classification via pose estimation experiment and obtain decent results. The second data capture was performed in TPG facilities from indoor environment of the shuttle as a real-environment in Geneva. We obtained 29 video sequences with 46,127 frames, demonstrating real conditions in an autonomous vehicle at the TPG depots. The merging of these two datasets led to substantially better results and allowed to perform more experiments. We performed additional evaluation using samples from NTU-RGB (without depth information) dataset, which helped to access the performance on unknown data and fine tune the model to generalize. In addition, samples from P-REACT and UCSD datasets were used to verify the results and implement the spatiotemporal autoencoder. Most of the collected data were annotated by VLabel, a cross-platform utility specifically developed for this purpose (Figure 2).

This tool was designed to improve productivity as it automates many procedures related to the time-consuming labeling process. The utility integrates the service’s critical components, such as the pose estimation backend and uses the same data format and structure. The simple and intuitive graphical user interface supports batch navigation, per frame or skeleton labeling and class switching.

## 4. Methodology

The following design principles were found to be important during the design of our system: (a) The solution should feature real-time detection when deployed on modern hardware available to the market and (b) the solution should support multiple camera types including wide-angle lens with higher fields of view (FoV). In this paper, three different approaches were implemented: (a) a Stacked Bidirectional LSTM Classifier, (b) a Spatiotemporal Autoencoder, and (c) a Spatiotemporal LSTM Classifier.

### 4.1. Stacked Bidirectional Lstm Classification via Pose Estimation

**Figure 3:**
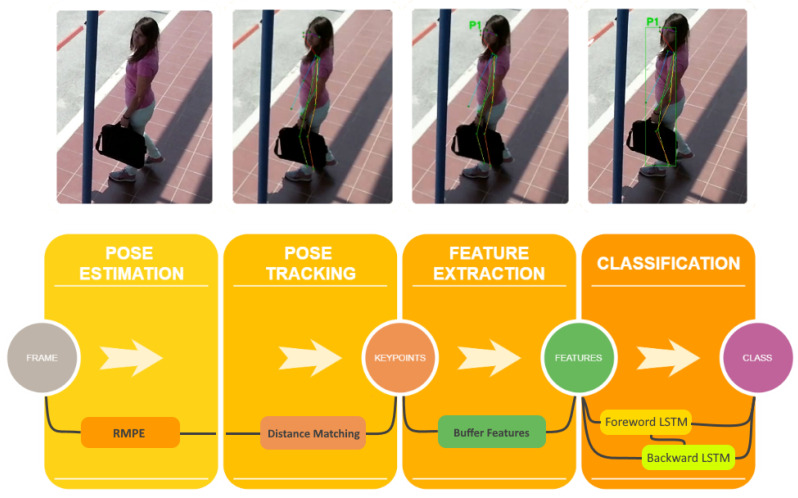
Pipeline of the pose classification.

The first approach consists of a stacked LSTM model as a classifier. An overview of the pipeline is depicted in Figure 3. Overall, the classification is performed in 4 stages: (a) In the first stage (Section 4.1.1), we apply pose estimation techniques to obtain skeleton keypoints. The generated pose proposals are refined by parametric pose non-maximum suppression to obtain the estimated human poses. (b) In stage two (Section 4.1.2), we perform tracking to match cross-frame poses and form pose flows. (c) In the third stage (Section 4.1.3), features are generated from the detected and tracked human body key-points and are being forwarded into the network (d), which classifies the action into normal or abnormal (Section 4.1.4).

#### 4.1.1. Pose Estimation

For the pose estimation stage we adopted the Regional Multi-person Pose Estimation (RMPE) by Fang et al. [27] with a pretrained VGG19 backend, a convolutional neural network model proposed by K. Simonyan et al. [28]. Based on extensive tests, VGG19 performed better in terms of accuracy at the cost of performance, which renders it ideal for generating training data. We also integrate more lightweight models, such as MobileNet v2, to be able to perform real-time inference on lower spec hardware. Moreover, our solution is based on abstract methods and can also utilize the OpenPose framework for generating keypoints based on PAFs and heatmaps. During the initial training phase, we intend to ameliorate the accuracy of the skeleton extraction for the training process and further perform data augmentation without sacrificing the data integrity. We generate noisy data with variable intensities, based on the extracted data from the backend, and we combine these data with the original ones as an augmentation technique. Extensive tests indicated that the model generalizes better and the accuracy improves. In this stage, 18 different human body keypoints are detected and the number of people in each frame is obtained.

#### 4.1.2. Tracking

In the tracking stage, we perform matching of cross-frame poses and form pose flows, using a real-time algorithm that is based on a distance matrix. In addition, a pose flow non-maximum suppression is applied, in order to reduce unnecessary pose flows and re-link temporal disjoint ones. This is an important step that associates poses indicating the same person, across multiple frames. We implemented a skeleton tracking algorithm, in order to meet the performance requirements of a real-time service. The algorithm is sorting the skeletons based on the distance between neck and image center, from small to large. Certain heuristics are taken into consideration, such as the position of the joints, the average height of the person and the height difference between frames. Height variation improves the ability of the algorithm to understand depth, since we are based on two dimensional input. Such parameters of the algorithms are optimized for in-shuttle space and fine-tuned to specified weights based on the camera calibration.

**Figure 4:**
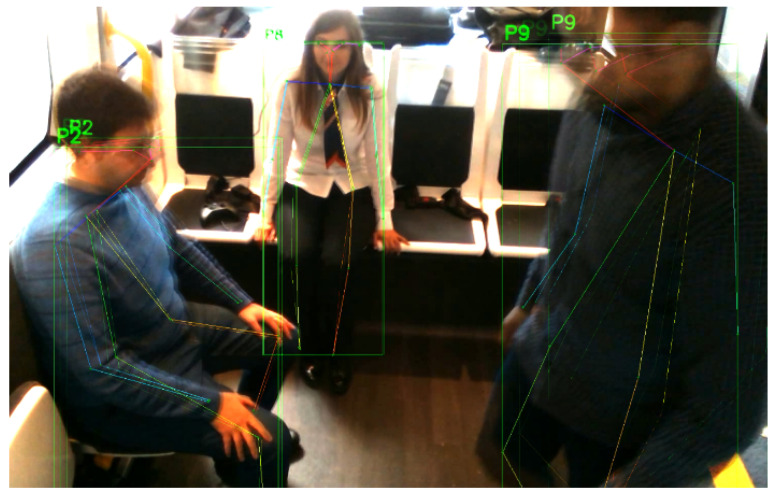
Skeleton matching across two subsequent frames (blended). Notice that the passenger ID, highlighted in green at the left of each bounding box, is the same across the frames.

A skeleton near center will be processed first and be given a smaller human ID. Later on, each skeleton’s features will be matched based on its previous and current frame. The distance matrix (or cost) between the skeleton joints is the main criterion for the matching function. Skeletons with the less distance are matched between the frames and are given the same ID (Figure 4).

**Figure 5:**
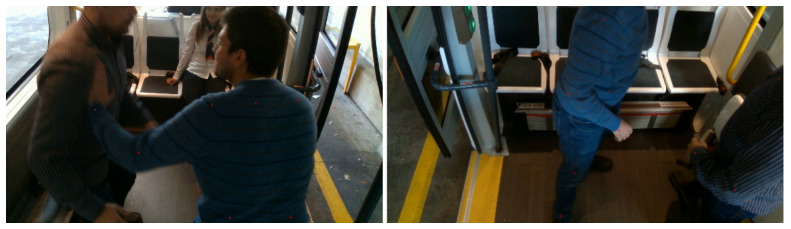
Examples indicating the restricted field of view of the camera sensor.

In some cases, the skeleton detection framework might fail to detect a complete human skeleton from the image due to the camera’s restricted field of view in the autonomous vehicle. Two examples of such situations are highlighted in Figure 5, where the cameras are not positioned properly due to the space constraints in the shuttle. This is a fundamental issue which can be mitigated either using sophisticated preprocessing or a wide angle camera lens, as we describe later on.

Events of occlusion can also cause missing joint positions, which should be filled with some values in order to maintain a fixed-size feature vector for the following feature classification procedure. To address this issue we fill in a joint’s position based on its relative position in the previous frame with respect to the neck. We also evaluated some other options with worse results: (a) Discard this frame. However, the algorithm would never be able to detect the action when the person is standing sideways and not facing the camera. (b) Fill in the positions with some value outside a reasonable range. Theoretically, when the classifier is strong enough, this method could work.

**Figure 6:**
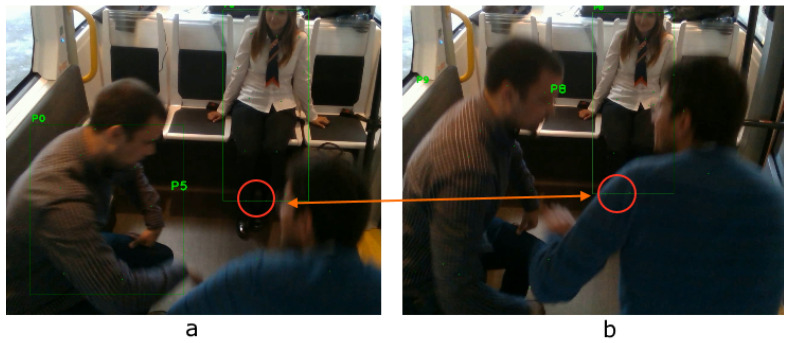
Despite the occlusion, the two leg joints of the girl are being approximately reconstructed (**b**) by their relative location in the previous frame (**a**).

An example of joint reconstruction is illustrated in Figure 6. However, we noticed that the classifier’s performance was degraded in some test cases. After extensive tests, we found that a previous joint position might be missing too, being replaced by the estimation of our algorithm. This led to “stuck” joints across various frames and confused both the tracker and the classifier. To overcome this issue, we are using a default “idle” pose as an example for our algorithm. When a previous joint is missing, the value being replaced is relative to the default “fallback” pose. We chose a person sitting as the default, because it is the most common for the passengers in the AV.

#### 4.1.3. Feature Extraction

Regarding the feature extraction stage, the detected and tracked human body keypoints are converted into features and “fed” into the LSTM neural network. In this stage, we also perform the removal of all the joints on head, since most of the actions do not involve much head movement, thus, the head’s position helps a little for the classification. For extracting features, we store every person’s skeleton data into a circular (ring) buffer double-ended queue (deque) of *N* frames (window_size) into the feature generator class. Afterwards, a fixed-feature vector will be constructed using the aforementioned skeleton data.

**Figure 7:**
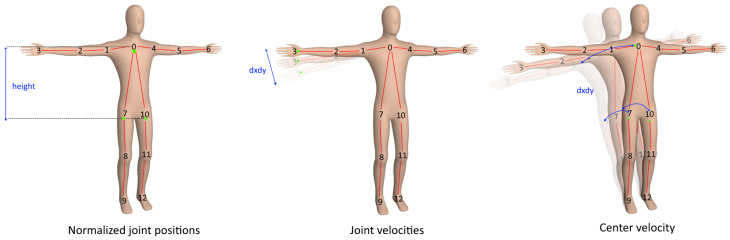
Representation of the extracted features. Joint positions (normalized), velocity, and body velocity are used as features for the classification.

The feature vector consists of features described in Table 2 and depicted in Figure 7, such as a direct concatenation of joints positions, the average skeleton height for normalizing joint positions, velocity of the joints, and the body’s center.

We consider the buffer as invalid if the newest appended skeleton does not contain at least the neck (Point 0) or one of the thigh bones (Point 7 or 10), as the height of the skeleton (used for normalizing features) cannot be calculated. The feature extraction process occurs when the buffer is full.

**Figure 8:**
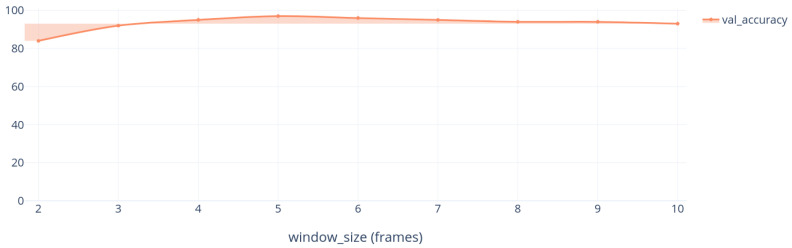
Performance evaluation across different buffer sizes. A buffer size of 5 frames achieved the best accuracy on the evaluation test.

The number of *N* frames for the feature generation along with the evaluation accuracy are depicted in Figure 8. A buffer size (window_size) of 5 frames achieved the best accuracy on the evaluation test. Higher values may result in lower accuracy as the tracker occasionally fails to consistently track people when the shuttle is overcrowded.

#### 4.1.4. Classification

The model is capable of binary or multi-class softmax classification. It contains three hidden layers of size (32×64) with the rectified linear unit (ReLU) activation function. The Bidirectional LSTM layers, as illustrated in Figure 3, connect two hidden layers of opposite directions to the same output. With this form of generative deep learning, the output layer can get information from past (backwards) and future (forward) states simultaneously. The model is trained end-to-end and regularized in such a way that helps with both distillation of the most compact profile of the normal patterns in the training data and detection of the abnormal events.

**Figure 9:**
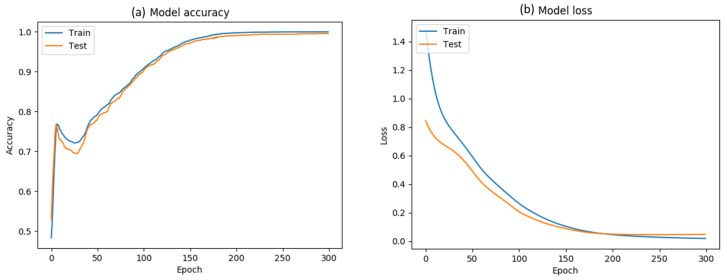
Model performance evaluation. (**a**) Train/test accuracy and (**b**) train/test loss metrics.

The evaluation performance of our model is depicted in Figure 9. We split the data with a 70-15-15 train/validation/test ratio and after 300 epochs, our model achieves a 99.6% accuracy. The time cost for feature extraction and classification is less than 50 ms per frame for the classifier, since the model is relative shallow.

### 4.2. Spatiotemporal Autoencoder

The second approach is based on the principle of frames’ dissimilarity in case of an abnormal event. Our goal, inspired by [29], is to train a spatiotemporal model consisting of the spatial and the temporal feature extractors. The combination learns spatiotemporal patterns of the frames’ input sequence. Therefore, in order to shrink the reconstruction error between the reconstructed input and output frame sequences, we trained our model using frame sequences of regular events. After our model’s proper training, we expect to have low reconstruction error in a normal video volume as opposed to a video volume containing abnormal scenes. Finally, the proposed framework will be able to detect the occurrence of an abnormal event, by thresholding on the error produced for each testing input volume.

#### 4.2.1. Architecture

There are two stages that form an autoencoder: encoding and decoding. Autoencoders set the number of encoder input units to be less than the input; thus, they were first used to reduce dimensionality. Usually, unsupervised back-propagation is used for training, helping the reconstruction error of the decoding results from the original inputs to decrease. Generally, an autoencoder can extract more useful features when the activation function is non-linear rather than some common linear transformation methods, such as Principal Component Analysis (PCA).

#### 4.2.2. Spatial Convolution

In a deep CNN, the main objective of convolution is to extract information from the input frame. The convolution process maintains the spatial relations of pixels by using kernels to extract low level features. In raw mathematics, the convolution operation performs dot products across filters of partial input. Supposing an nxn input layer, followed by a convolutional layer, then if we use an m×m filter *W*, the output size will be (n−m+1)×(n−m+1). Through the training stage, a CNN learns the values of these filters by itself, although some parameters such as the filter size and the number of layers still need to be defined. The larger the number of filters used, the more information that gets extracted and the better the network generalizes. Yet, there is a trade-off and balance is a critical factor when it comes to the number of filters used, as more filters would impact the performance negatively and require more resources.

#### 4.2.3. Preprocessing

At this stage, our task is to transform the raw information into the correct input for our model. To do so, we perform alignment of each frame extracted from the raw videos, followed by a resize operation to the resolution of (227×227). In order to verify the same scale across the frame pixels, we normalize them between 0 and 1 and we subtract the global mean image. To compute the mean frame, we average the pixel values across multiple frames in the dataset. The next step includes a conversion to grayscale in order to reduce dimensionality. We also evaluate various methods such as the Gunnar–Farneback method [30] for calculating optical flow, mixture of Gaussians in order to separate background [31] and frame subtraction via the absolute difference of the frames. Afterwards, the preprocessed frames are normalized to zero mean and unit variance. The input of our model is a buffer of 10 frames with multiple strides. Following [29] practice, we perform data augmentation in the temporal dimension. Moreover, the use of strides improves the performance and the generalization of our model on variable frame rates. As an example, the stride-1 sequence consists of frame numbers 1, 2, 3, 4, 5, 6, 7, 8, 9, 10, whereas the stride-2 sequence contains frame numbers 1, 3, 5, 7, 9, 11, 13, 15, 17, 19, and stride-3 sequence would contain frame numbers 1, 4, 7, 10, 13, 16, 19, 22, 25, 28.

#### 4.2.4. Feature Learning

**Figure 10:**
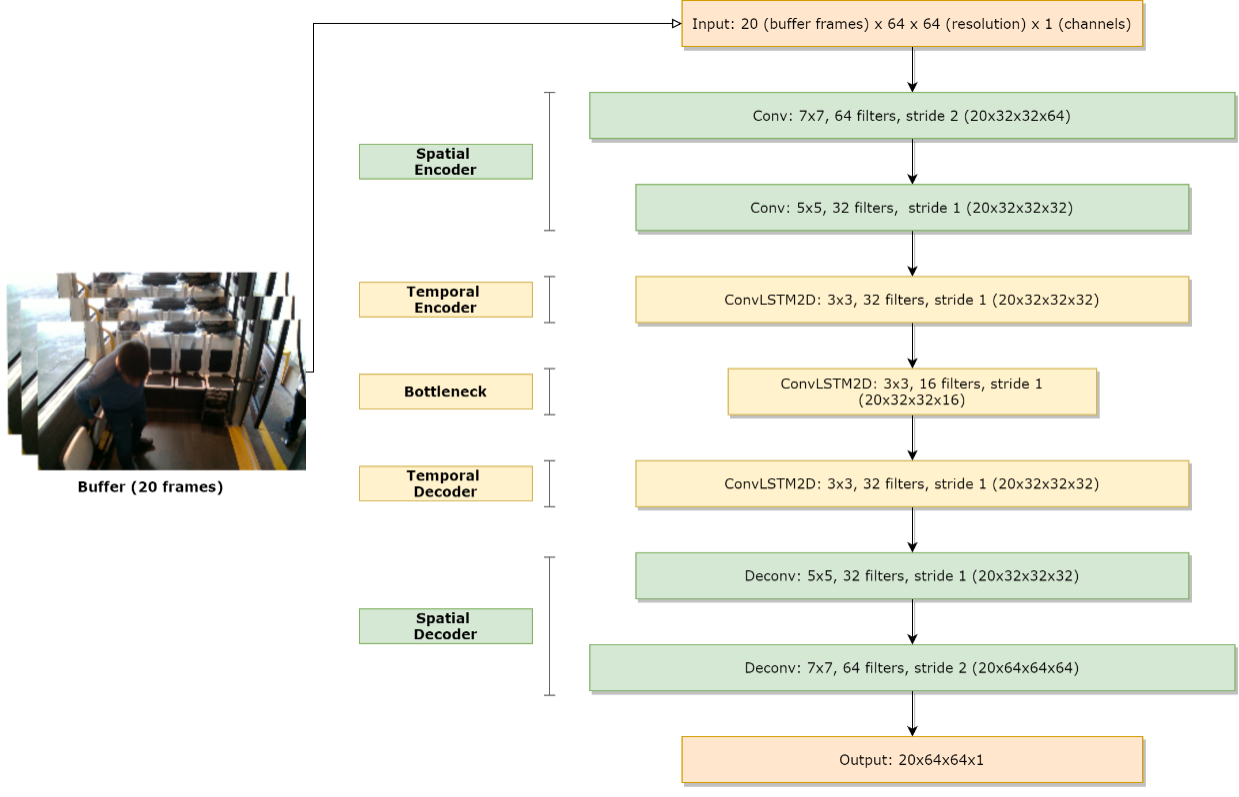
Model architecture of the autoencoder: The first two convolutional layers are spatial encoders, followed by temporal encoder and decoder. Between them, a ConvLSTM with reduced filters is used as a bottleneck to eliminate non useful information. At the last two layers we perform spatial decoding, reconstructing the input image to the same format.

In order to learn the regular events in training data, we introduce a spatiotemporal autoencoder. Our model consists of (a) a spatial autoencoder in order to obtain spatial features and (b) a temporal encoder-decoder to learn temporal features of the encoded spatial features. In particular, the spatial autoencoder consists of an encoder and a decoder that are composed of two convolutional and transpose convolutional layers, respectively, whereas the temporal encoder is comprised of three convolutional LSTM layers, as depicted in Figure 10. Convolutional layers perform excellent in object detection, whilst the LSTM model is applied for learning sequences and modeling time-series and has shown superior performance in applications such as speech translation and handwriting recognition.

#### 4.2.5. Regularity Score

**Figure 11:**
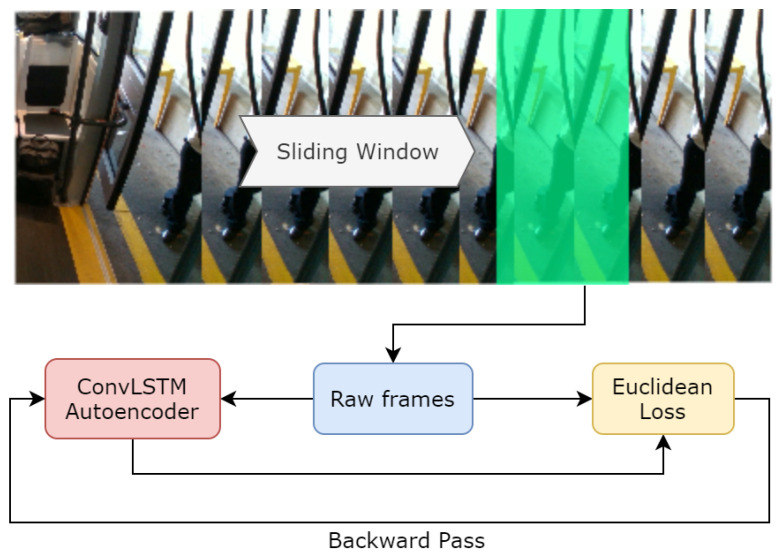
Preprocessing using a sliding window of 10 frames and training pipeline. Euclidean loss is used to learn regularity.

We define the reconstruction error as the Euclidean distance across an input and a reconstructed frame (Figure 11). Specifically, the following equations describe the reconstruction error, where *t* denotes the frame in a sequence:

(1) $$e\left(t\right)={∥x\left(t\right)-f_{W}\left(x\left(t\right)\right)∥}^{2}$$

where $f_{W}$ is the learned weights by the spatiotemporal model. The irregularity score $s_{a}\left(t\right)$ is calculated by scaling between 0 and 1. Eventually, the regularity score $s_{r}\left(t\right)$ can be derived by subtracting the reconstruction score from 1:

(2) $$s_{a}\left(t\right)=\frac{e\left(t\right)-e{\left(t\right)}_{\min}}{e{\left(t\right)}_{\max}}$$

(3) $$s_{r}\left(t\right)=1-s_{a}\left(t\right)$$

Once the model is trained, its performance can be evaluated by feeding in testing data and checking whether it can detect abnormal events while maintaining a low false alarm rate. For a better comparison, we calculated the regularity score for all frames employing the same formula as [29]. Our only difference is that the trained model is of another kind.

#### 4.2.6. Thresholding

**Figure 12:**
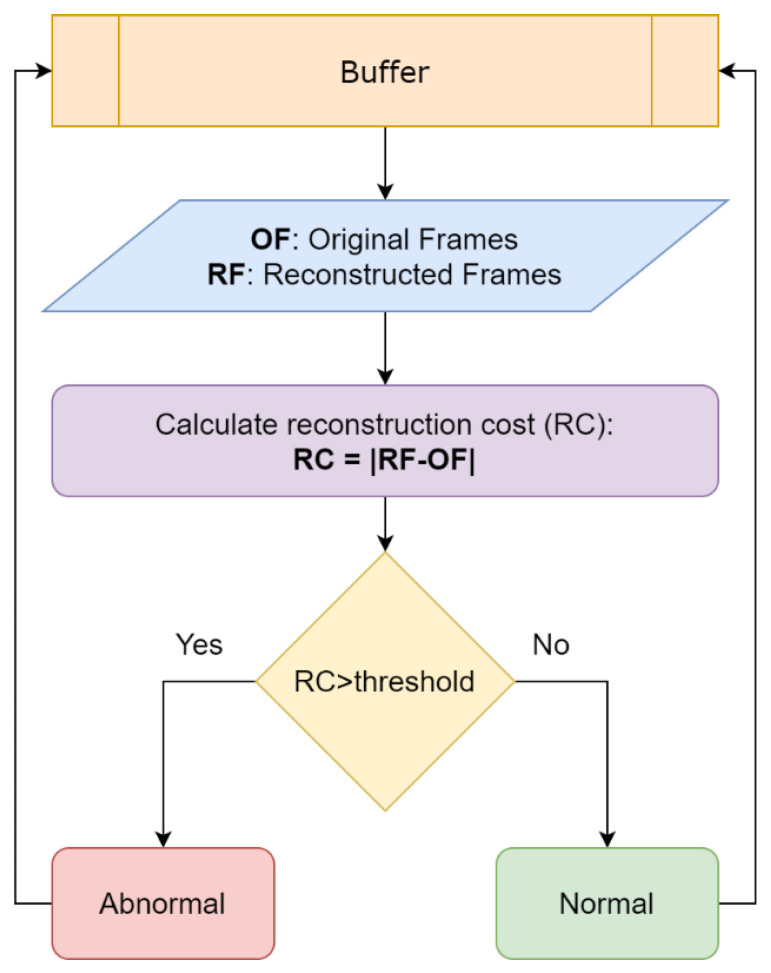
Decision flow: A high reconstruction cost between OF and RF indicates an abnormal event.

**Figure 13:**
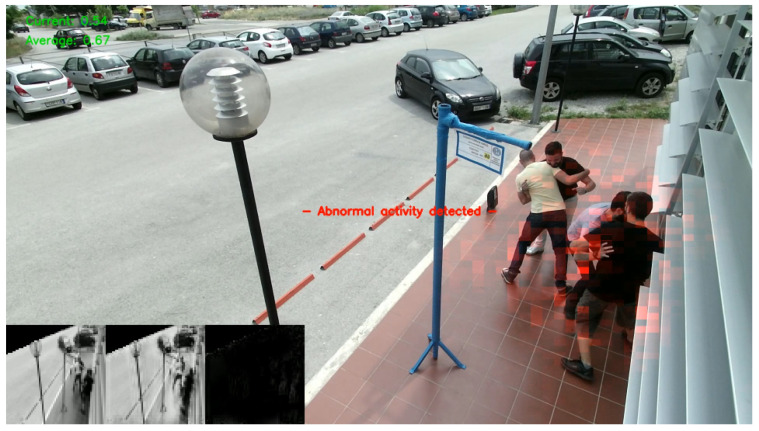
Outdoor group fighting scenario on a simulated bus stop. Green metrics at top-left indicate the current and the average regularity score. A lower regularity score indicates that the predicted reconstruction is not accurate, since our model did not learn such an event. Note that the current score is much lower than the average, triggering an abnormal notification.

Using a threshold on the reconstruction error, we are able to determine whether a video frame is normal or abnormal (Figure 12). A predefined threshold is not a robust method since our solution should operate in real-time and support multiple camera sensors as we mentioned earlier in the design principles. A fixed threshold value can alter the sensitivity of the event detection, rendering it inappropriate in some scenarios. In addition, a wrong threshold can prevent the detection of certain abnormal events or produce false positives. In order to solve this issue, we introduce a variable thresholding technique in order to find the optimal value in real-time. The initialization procedure now includes a “warm-up” session, in which we aggregate the individual regularity score of each frame. During that session, no detections are performed, as we consider the events as regular. As the buffer continues to fill, we are able to calculate the average reconstruction error and provide a threshold value tailored to the specific conditions. Figure 13 indicates an abnormal scenario with the aforementioned metrics.

### 4.3. Hybrid LSTM Classification

**Figure 14:**
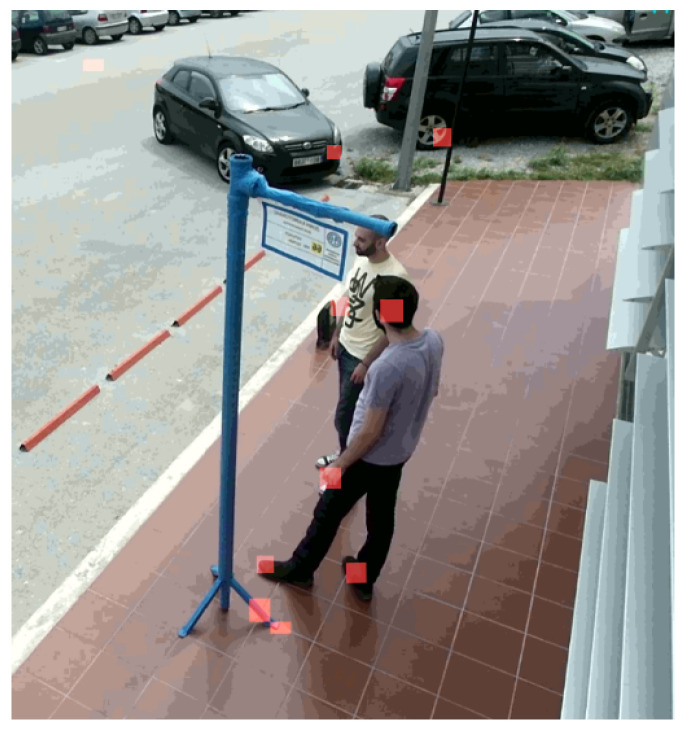
Example of a prediction with a lower regularity threshold.

**Figure 15:**
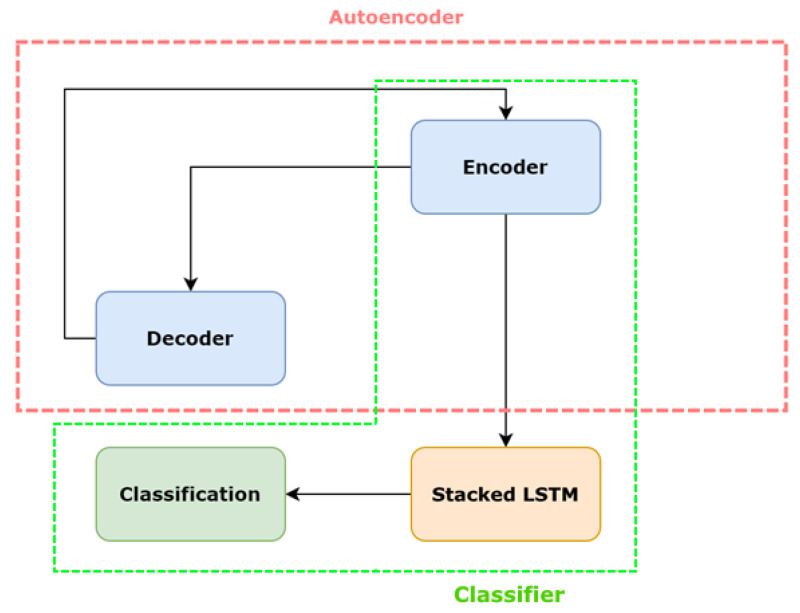
Model architecture of the hybrid model. The red container contains components of the previous autoencoder approach. The green components indicate the new hybrid model which acts as a classifier.

Even if we train our model on thousands of data, we will still get some false positives in certain occasions. As a result that we are able to manually shift through the anomaly outputs and flag some of them as false positives, we can let the previous autoencoder neural network model act as a high recaller. We employ semi-supervised learning by decreasing the threshold, so that we can detect the majority of true anomalies (high recall), as well as other false positives (low precision) (Figure 14). To achieve the semi-supervised approach, we designed a new model which includes the previous encoder and an LSTM which acts as a classifier as depicted in Figure 15.

In real-time inference, the anomalies predicted by the autoencoder neural network model (mentioned as high recaller) are sent through the false positive reduction model (hybrid model). Combining these two neural networks, should provide a deep neural network model that offers both high precision and high recall. Furthermore, we no longer depend on a prone to error threshold value for classifying events. We refer to this technique as a weak supervision method.

**Figure 16:**
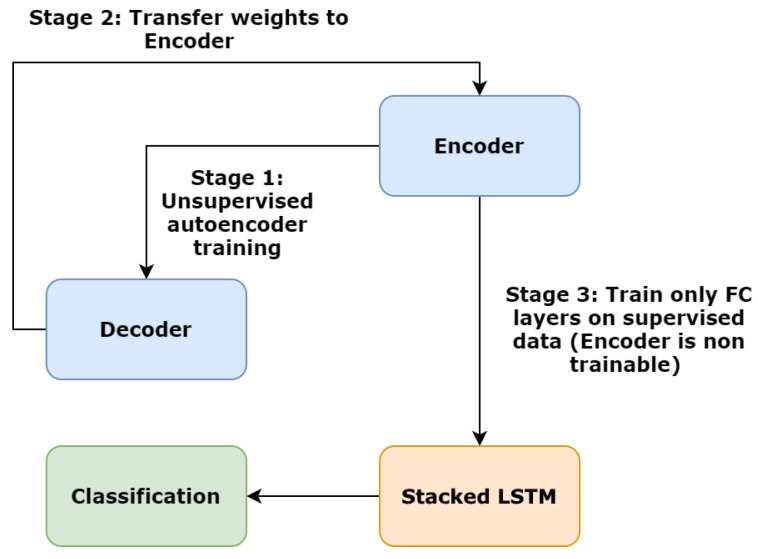
The three stage pipeline of the hybrid training procedure consists of an initial unsupervised training, followed by transfer learning and retraining of the stacked LSTM Classifier.

The training process of the new experiment consists of 3 stages, as Figure 16 illustrates: At first, the autoencoder model (encoder + decoder) is being trained in an unsupervised manner to learn regularity. In the second stage, the encoder weights are transferred to the hybrid model. The new hybrid model consists of an additional stacked LSTM layer, which acts as a classifier. The encoder‘s layers are marked as non-trainable. Finally, we perform supervised training only on the LSTM Classifier.

**Figure 17:**
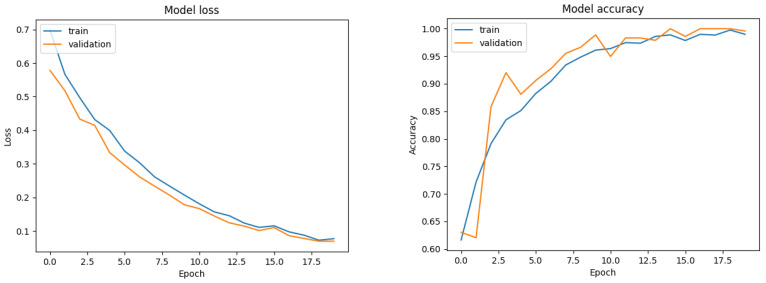
Train/Val loss and accuracy of the hybrid classifier, over 20 epochs.

We trained the hybrid model for 20 epochs, using an 64/16/20 train/validation/test ratio. The loss and the accuracy are depicted in Figure 17. The final model accuracy is 98%.

## 5. Experimental Results

The environment for the experiments consist of an Intel(R) Xeon(R) CPU E5-2630 v4 @ 2.20 GHz with a TDP rated to 85 W (Thermal Design Power). The operating system is the Red Hat Enterprise Linux Server 7.4 × 86_64. The system utilizes 2 × Tesla K40 m GPUs for acceleration. The Total Graphics Power (TGP) of each GPU is rated to 235 W. The system supports most off-the-shelf cameras. The dimensions and the weight of the sensors vary and the mounting is performed manually. In terms of resolution, the sensor provides 1080p (FullHD) resolution images (1920 × 1080 pixels) at 24 frames per second and the operating temperature range applies (e.g., 5 to 35 degrees Celsius). Sensors are powered via the USB 2.0 and Power over Ethernet (PoE) connections. The RGB video stream is set to 24-bit FullHD 1080p resolution. The data rate is configured at 30 Hz but can be adjusted on demand. The data stream is continuous and can be also acquired on request. The API and the SDK of the camera are flexible enough to acquire the images as soon as they are required on the aforementioned maximum data epoch rate. As Table 3 indicates, depending on the perspective of the final camera setup and the technical specifications of the lens/sensor, different techniques are applied.

### 5.1. Video Analysis

**Figure 18:**
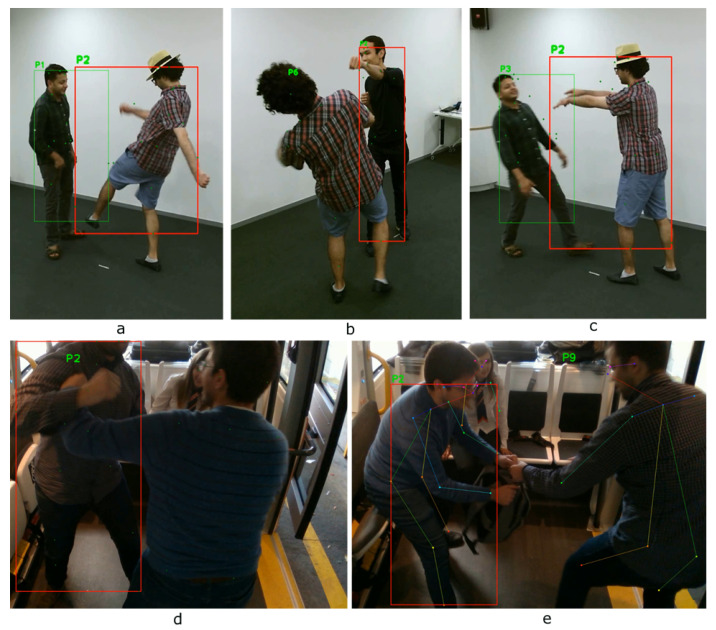
Evaluation on test data: (**a**–**c**) Abnormal event detection (violence/passengers are fighting) using different camera angles from the NTU-RGB dataset. (**d**,**e**) Detection of fighting/bag-snatch real-world scenarios inside the shuttle.

**Figure 19:**
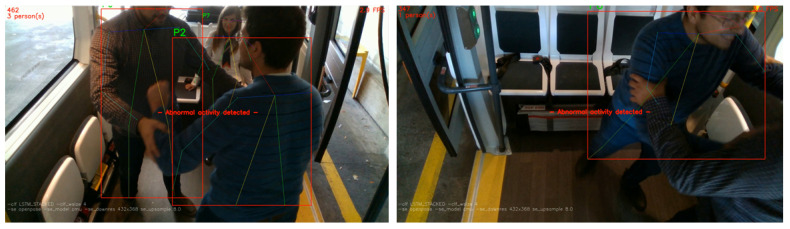
Evaluation on multiple camera angles, excessive occlusion, and partial presence.

**Figure 20:**
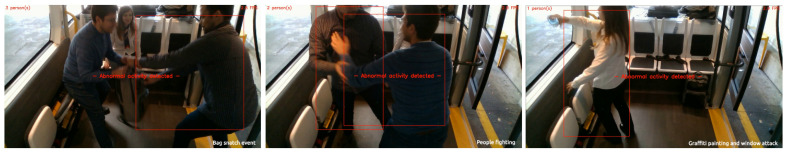
Evaluation across various scenarios (**left** to **right**): Bag-snatch, fighting, and vandalism.

**Figure 21:**
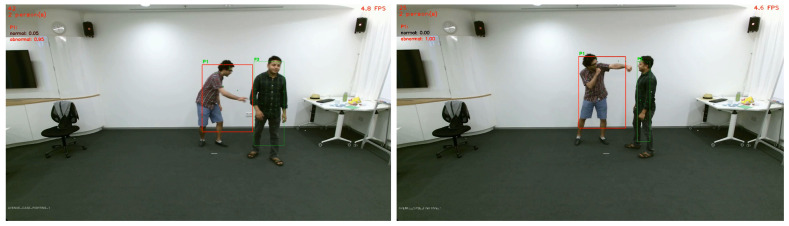
Additional evaluation on the NTU-RGB dataset. Metrics at the top-left depict the prediction scores for the P1.

We tested our pose classification approach on the NTU RGB+D dataset [26] (Figure 18a–c) and on the TPG dataset captured by CERTH inside the AV’s shuttle (Figure 18d,e). In the images, there are various debugging layers enabled, such as skeleton points, lines, tracker ID, and bounding boxes of each detection. The predicted result is marked as green, when the classifier indicates it as “normal” and red when “abnormal”, correspondingly. So far, we did not include NTU dataset samples in our training set, so it is safe to assume that our model can generalize across different people, view angles, and events. Figure 19, Figure 20 and Figure 21 depict the aforementioned conditions and use cases.

The following Equations (4)–(7) describe the precision, recall (sensitivity), F1-Score, and accuracy metrics illustrated in Table 4, while true positives (TP)/negatives (TN) are the correctly predicted positive/negative values and false positives (FP)/negatives (FN) are the incorrect predictions. In addition, Table 4 contains the classification report of the pose classifier on the test data.

(4) precision=TP/TP+FP

(5) recall=TP/TP+FN

(6) F1=2∗(Recall∗Precision)/(Recall+Precision)

(7) accuracy=TP+TN/TP+FP+FN+TN

Based on a recent survey by Zhang et al. [32], we can compare with similar methods that are evaluated on the NTU-RGB dataset. In addition, the NTU-RGB features 60 classes and some of them fit to our “abnormal” class. This is the main reasons for selecting this dataset. For the sake of completeness, we evaluated our model both in the NTU-RGB-D and our dataset. As Table 5 illustrates, our method performed relatively good with 71.4% accuracy on the NTU-RGB-D, despite the lack of depth information. Our augmentation techniques along with the tracking algorithms contributed to this result. In our dataset, which contains data from CERTH labs, TPG depot, P-REACT, and some samples of the NTU-RGB-D dataset, our approach performed exceptionally good with 99.6% accuracy.

**Figure 22:**
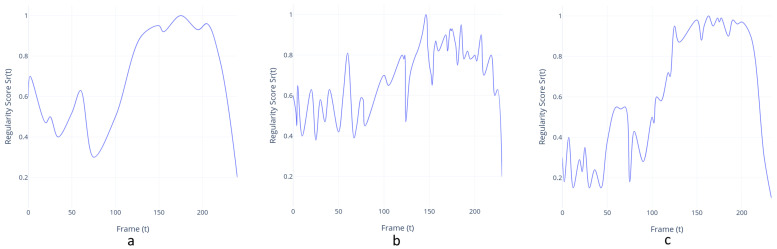
Regularity curves (**top**-**left**) with a bag-snatching scenario using three preprocessing methods: (**a**) MOG2 background subtraction, (**b**) frame subtraction (absdiff), (**c**) Farneback optical flow. All methods managed to detect the abnormal events in 80th and 250th frame. MOG2 performed better in terms of stability and achieved more consistent results on passenger boarding and disembarking.

Moving on to the autoencoder-based techniques, we trained our models on the P-REACT and TPG datasets. For the sake of completeness, we also train and compare our model with similar methods on the UCSD-Ped1 dataset [13]. The approach achieved frame-level area under ROC curve (AUC) of 85.2 and 18.9 Equal Error Rate (EER) in our dataset, as shown in Table 6. Furthermore, our approach achieves an AUC/EER ratio of 88.2/13.1 on the UCSD-Ped1 dataset even though the preprocessing and parameters are not tailored to the specific application. Regularity curves of a bag snatching scenario are illustrated in Figure 22.

**Figure 23:**
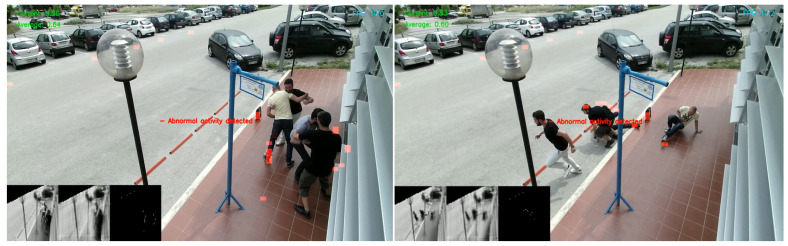
Experimental outdoor evaluation with a fighting scenario on a simulated bus stop. Red regions represent abnormalities in the frame.

In order to visualize the predictions, we provide the preprocessed input frame for the current moment at the bottom-left. The frame is resized from 64 × 64 and grayscale and a mask overlay is obscuring the out-of-interest areas (road). At the right next to the input frame, the resized output of our model is shown. The third mini-frame demonstrates only the significant differences between the input and the output frames with white pixels which is then merged above the original frame for demonstration purposes. Figure 23 illustrates the predicted pixel-level anomalies of an outdoor fighting scenario in a simulated bus stop.

**Figure 24:**
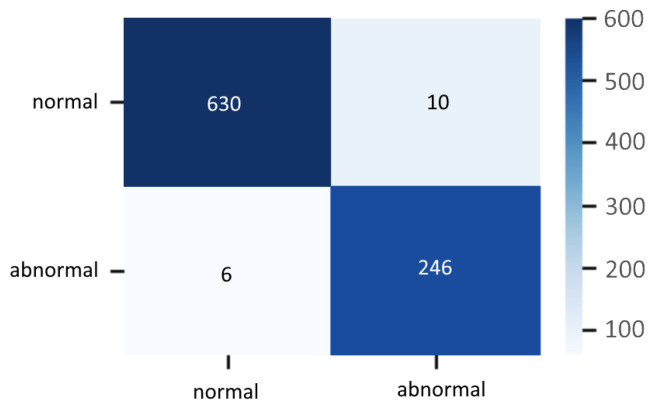
Confusion matrix of the hybrid classifier on the test data.

Experimental results on test data are enlisted in Table 7, which contains the classification report of the hybrid classifier. The confusion matrix is depicted in Figure 24.

### 5.2. Performance/Energy Analysis

**Figure 25:**
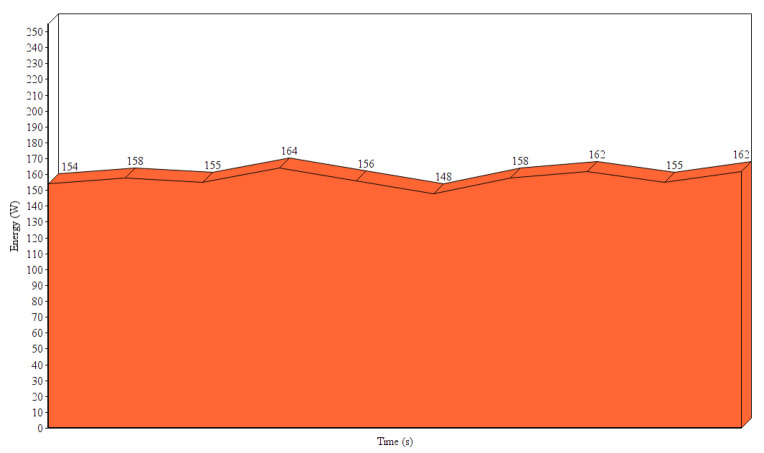
NVIDIA Tesla K40 m GPU energy consumption on full usage (avg. 13 fps).

Since we are targeting an autonomous platform, we need to evaluate the efficiency of this system in terms of performance and power requirements. We obtained the approximate GPU power consumption (Figure 25) using the driver tools provided by Nvidia. We estimated a maximum of 100 W overhead by the CPU and other components of the system. In general, it would be safe to assume that our system’s energy requirements on full speed peaks at 300 W in total at the aforementioned hardware. The maximum framerate achieved on a single Tesla K40 m is about 13 fps (77 ms per frame). Experiments indicated that locking the framerate at a slightly lower value (10 fps) yields much more stable performance and reduces the total power consumption.

## 6. Conclusions

In this research, various techniques were implemented for the timely, accurate, robust, and automatic detection of abnormal events. Three different approaches were presented for anomaly detection specifically tailored to the perspective of the final camera setup and the technical specifications of the lens/sensor (Table 3). Pose classification is a supervised approach that classifies the extracted skeleton key points based on an annotated dataset. It can reliably detect various types of events but depends on a previous skeleton extraction and tracking process, which may not be accurately feasible due to space and occlusion constraints. It does not depend on the camera setup (but it is less effective on top-down cameras with a wide field of view). Although the LSTM Classification via pose estimation performs generally better, it cannot be applied with satisfactory results in the existing in-shuttle camera. Autoencoder based solutions were developed to prevent excluding this possibility, since autoencoders do not depend on preprocessing to obtain pose information and they extract features by learning regularity. The convolutional LSTM autoencoder (unsupervised) extracts spatiotemporal features from a video sequence, in order to learn regularity. It can detect abnormal events and activities and depends on the camera setup but due to its unsupervised nature it can be trained over-time and self-improve. It is ideal for crowded areas and static camera setups. In Hybrid classification (semi-supervised), the aforementioned encoder acts as a high recaller, and the anomalies are sent through a false positive reduction model (hybrid model). This combination provides a deep neural network with high recall and high precision. The proposed solution achieves up to 99.6% accuracy on our test data, supports multiple camera types and is capable of real-time inference on modern hardware setup. It is expected to be useful in the timely detection of petty crimes incidents and other abnormal events, according to standard procedures adopted by the transport operator.

## Figures and Tables

**Table 1:** Methods comparison.

Method	Strengths	Drawbacks
[13]	crowded scenes	offline, low accuracy
[14]	high accuracy	requires data from wearable sensors
[15]	robust, high accuracy	requires data from additional sensors
[16]	supervised/unsupervised learning	multiple camera types, angle support
[19]	high performance	low accuracy, events of occlusion
[20]	robust, high accuracy	requires depth, acceleration data
proposed	flexible, high accuracy, multiple FoV, positions, angles	requires a lot of data, fine tuning

**Table 2:** A description of the features generated in the feature extraction stage for the action classification.

Feature	Description
Xs	A direct concatenation of joints positions of N frames.
H	Average height (Neck to Thigh length) of N frames. Used for normalization.
X	Normalized joint positions [Xs − mean(Xs)]/H
Vj	Velocities of the joints {X[t] − X[t − 1]}
Vc	Velocity of the center {sum(Xc[t] − Xc[t − 1])} (10× weight)

**Table 3:** Technical specifications of the equipment used in the experimental environment.

Solution	Pose Classification	Regularity Learning	Hybrid Classification
Camera	oCam 5MP sensor	AXIS M3046-V	AXIS M3046-V
Power Supply	USB 3.0	PoE	PoE
Host	In-shuttle	In-shuttle	In-shuttle
Power Supply	500 W (max)	500 W (max)	500 W (max)

**Table 4:** Precision, recall, and F1-Score metrics for the two classes.

Class	Precision	Recall	F1-Score	Support
Normal	0.99	0.99	0.99	6040
Abnormal	0.93	0.95	0.94	1335
Accuracy			0.99	7375
Macro avg	0.96	0.97	0.96	7375
Weighted avg	0.99	0.99	0.99	7375

**Table 5:** Experimental comparison with human action recognition methods based on the recent survey by Zhang et al. [32]. The proposed method does not utilize depth information from the NTU-RGB-D dataset.

Method	Year	NTU-RGB-D	Custom Dataset
[33]	2020	91.5%
[34]	2020	90.3%
[35]	2018	73.4%
[12]	2018	30.7%
[26]	2016	62.93%
[36]	2016	69.2%
[37]	2014	31.82%
Proposed	2020	71.4%	99.6%

**Table 6:** Comparison of area under ROC curve (AUC) and Equal Error Rate (EER) of different methods on the UCSD-Ped1 dataset [13]. Higher AUC and lower EER are better.

Method	Area under ROC Curve (AUC)	Equal Error Rate (EER)
[29]	81.0	27.9
[38]	89.9	12.5
[39]	72.7	33.1
[40]	77.1	38.0
[13]	66.8	40.0
[41]	67.5	31.0
Proposed	88.2	13.1

**Table 7:** Precision, Recall, and F1-Score metrics on the test set of the hybrid classifier.

Class	Precision	Recall	F1-Score	Support
Normal	0.98	0.99	0.99	640
Abnormal	0.98	0.96	0.97	252
Accuracy	-	-	0.98	892
Macro avg	0.98	0.97	0.98	892
Weighted avg	0.98	0.98	0.98	892
